# Degraded stimulus visibility and the effects of perceptual load on distractor interference

**DOI:** 10.3389/fpsyg.2013.00289

**Published:** 2013-05-29

**Authors:** Yaffa Yeshurun, Hadas Marciano

**Affiliations:** ^1^Department of Psychology, University of HaifaHaifa, Israel; ^2^Institute of Information Processing and Decision Making, University of HaifaHaifa, Israel

**Keywords:** perceptual load, stimulus visibility, attentional selectivity, distractor interference, task difficulty, sensory load

## Abstract

In this study we examined whether effects of perceptual load on the attentional selectivity are modulated by degradation of the visual input. According to the perceptual load theory, increasing task difficulty via degradation of stimulus visibility should not alter the typical effect of perceptual load. In previous studies only the target was degraded, resulting in increased distractor saliency. Here we combined manipulation of perceptual load with a more systematic degradation of visual information. Experiment 1 included five conditions. Three conditions involved low perceptual load + contrast reduction of: (A) only the target; (B) only the distractor; (C) both target and distractor. The other two conditions included non-degraded stimuli with low or high perceptual load. In Experiment 2 visibility degradation was established via manipulation of exposure duration. It included two exposure durations—100 and 150 ms—for each load level (low vs. high). The results of both experiments demonstrated reliable distractor interference of a similar magnitude with both degraded and non-degraded stimuli. This finding suggests that task difficulty, when manipulated via degradation of stimulus visibility, does not play a critical role in determining the efficiency of the attentional selectivity. However, contrary to the predictions of the perceptual load theory, in both experiments distractor interference emerged under the high load condition. In Experiment 2 the high-load interference was of the same magnitude as that of the low load condition. This high-load interference is not due to the presence of a mask (Experiment 3) or a mixed design (Experiment 4). These findings suggest that perceptual load may also play a lesser role in attentional selectivity than that assigned to it by the perceptual load theory.

## Introduction

The perceptual load theory (e.g., Lavie, 1995; Lavie and Cox, 1997; Maylor and Lavie, 1998) offers a theoretical account for the fact that in some cases the attentional selectivity seems too high (e.g., Mack and Rock, 1998; Mack et al., 2002), while in other cases the attentional selectivity seems too low (e.g., Eriksen and Eriksen, 1974; Theeuwes, 1992). It suggests that perceptual load, defined as the need to carry out further perceptual operations or apply the same operation to additional units, is the critical factor that determines the extent to which non-attended information is processed. According to the theory, as long as capacity limitations were not met, perceptual processing proceeds automatically on all stimuli, relevant or not. Once the capacity exceeds its limitations, irrelevant information can no longer be processed. When the relevant information imposes a high load it exhausts the available processing capacity and in turn the processing of irrelevant information is prevented. To support the theory, Lavie and Cox (1997) varied the load by changing the similarity between a target and non-target letters and the heterogeneity of the non-target letters. The target, “N” or “X,” was presented in one of six positions on an imaginary circle. The other five positions were occupied by either other heterogeneous letters (H, M, K, Z, W) in the high perceptual load condition, or by five homogeneous “O's” in the low perceptual load condition. The task was to indicate whether there was an X or an N in the circle of letters while ignoring a peripheral distractor letter. The distractor was either compatible with the target, incompatible or neutral. A compatibility effect—incompatible reaction time (RT) minus neutral RT—was found in the low load condition but was absent in the high load condition. Hence, in accordance with the perceptual load theory, the low load condition resulted in an inefficient filtering out of distractors, while the high load condition resulted in an efficient filtering out of distractors. Similar results were found with different stimuli and manipulations of perceptual load (e.g., Handy et al., 2001; Lavie and Robertson, 2001; Bahrami et al., 2007; Brand-D'Abrescia and Lavie, 2007; Rorden et al., 2008; but see Khetrapal, 2010; Tsal and Benoni, 2010; Marciano and Yeshurun, 2011).

In this study we tested whether degrading the quality of the visual input will modulate these effects of perceptual load, as defined above by the perceptual load theory. One possible alternative explanation of previous findings suggests that the lack of compatibility effect in the high load condition is not due to the exhaustion of processing resources brought about by the high levels of load but simply to the fact that the task in this condition is considerably more difficult than that in the low load condition. Lavie and de Fockert's (2003) study was designed to disprove this alternative explanation. They based their rationale on the assumption that perceptual load is different from sensory limits. This assumption was inspired by Norman and Bobrow's (1975) distinction between data-limited and resource-limited processes. Data limits refer to limitation on the quality of the input while resource limits refer to limitation on resources available for the processing of the input. Applying more resources may not overcome data limitation. As mentioned above, according to the perceptual load theory, perceptual load can be operationalized in two different ways: either additional perceptual operations must be carried out (e.g., the processing of a target defined by feature vs. the processing of a target defined by a conjunction of features), or the same operations must be applied to more items (e.g., a manipulation of set size). The argument of the perceptual load theory is that the additional operations or items in the high load condition consume attentional capacity, thereby preventing the processing of the irrelevant information. However, making the task harder by increasing data limits without increasing perceptual load (as defined above) should not consume attentional capacity and therefore the irrelevant distractor should be processed and interference should be found. Thus, manipulating the sensory limits via degradation of target visibility (i.e., degrading the quality of the input) should increase general task difficulty but should not impose additional demands on the attentional resources, leaving available capacity for distractor processing. To test this hypothesis they compared the effects of perceptual load on distractor interference with the effects of various manipulations of sensory degradation of the target stimulus. They employed three manipulations of target degradation that involved three different combinations of the following: decreasing the size and contrast of the target, shortening the exposure duration, presenting masks after the target display that mask the target, and increasing the eccentricity of the target. The results of these experiments showed that although degrading the quality of the sensory input of the target increased RT, indicating that the degradation manipulation increased task difficulty, it did not reduce the interference of the irrelevant distractor. Irrelevant distractor interference was found in all the degraded target conditions and it was even greater than the interference in the non-degraded low load conditions. Such interference was not found in the conditions of high perceptual load. According to Lavie and de Fockert (2003), this pattern of results suggests that merely impairing the sensory input of a target stimulus is not equal to overloading the perceptual process. To load the perceptual processes one should add perceptual operations or more items to the task. Thus, Lavie and de Fockert concluded that while perceptual load decreases distractor interference, sensory degradation increases it.

However, Lavie and de Fockert (2003) only degraded the target. That is, only the target contrast and size was reduced; only the target positions were masked—there were no masks at the distractor positions; and only the target eccentricity was increased. Because the distractor in their study was not degraded at all, target degradation might have rendered the target less conspicuous in comparison to the distractor. It is possible, therefore, that the processing of the degraded target did require more resources but because the distractor was more conspicuous it nevertheless interfered with the response to the target (either because fewer processing resources were needed for it to cause interference due to its higher conspicuity, or because its conspicuity captured attention away from the target). If so, the assumption that perceptual load is different from sensory limits does not hold. The goal of the current study was to test in a more comprehensive way the effect that degrading the quality of the visual input may have on distractor interference. To that end, we combined manipulation of perceptual load with degradation of visual information in both relevant and irrelevant regions of the visual field. Specifically, in Experiment 1, we employed a similar load manipulation to that of Lavie and de Fockert and added three different conditions of degradation: (A) contrast reduction of both the target and distractor stimuli; (B) contrast reduction of the target stimuli alone; and (C) contrast reduction of the distractor stimuli alone. All three conditions involved low levels of load, and distractor interference under these degraded conditions was compared to that with two non-degraded conditions—one with low levels of load and the other with high levels of load. Experiment 2 included a load manipulation that is similar to that of Lavie and Cox (1997), and a different manipulation of visibility degradation. Specifically, we shortened the exposure duration of all the stimuli and compared this degraded condition with a condition that included the typical exposure duration. All possible target and distractor positions were masked. To foreshadow the results of both experiments, we found reliable distractor interference regardless of degradation manipulation. However, in contrast to the predictions of the perceptual load theory, reliable distractor interference was also observed in the high load condition. Experiments 3, 4 explored possible explanations for this latter finding.

## Experiment 1

**Figure 1:**
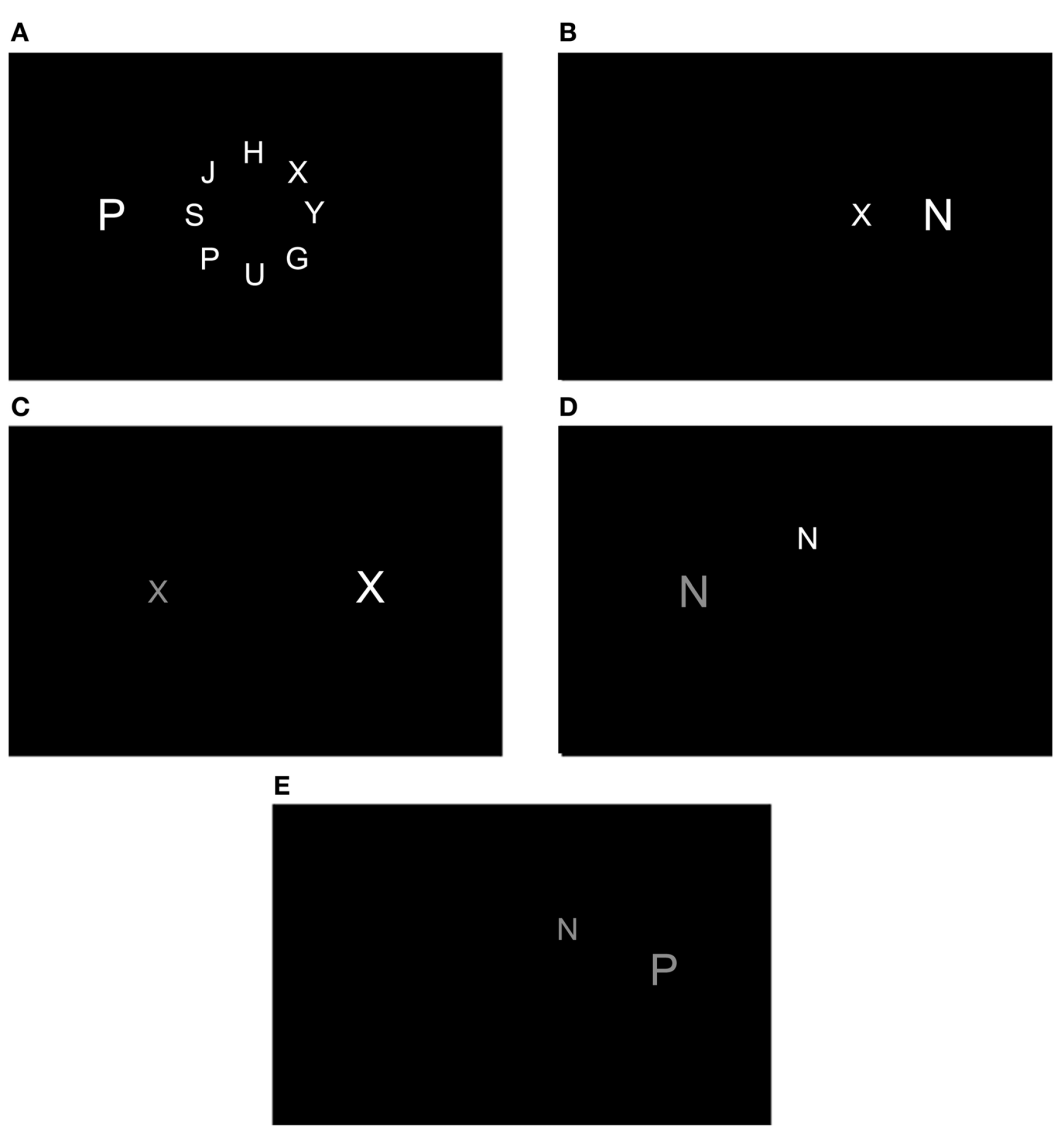
**The various conditions of Experiment 1: (A) High load, no degradation (HLND); (B) Low load, no degradation (LLND); (C) Low load, target degradation (LLTD); (D) Low load, distractor degradation (LLDD); (E) Low load, both target and distractor degradation (LLBD)**.

In this experiment we extended the degradation manipulation to also include non-relevant locations. To degrade the quality of the visual input we lowered the contrast of the stimuli in three different degradation conditions: (1) the contrast of both target and distractor was reduced; (2) the contrast of the target was reduced but not that of the distractor. This condition is similar to Lavie and de Fockert (2003); and (3) the contrast of the distractor was reduced but not that of the target. These degraded conditions involved low levels of perceptual load, and distractor interference (i.e., compatibility effect) in these conditions was compared to that of a fourth low load condition with the contrast of both target and distractor intact. Finally a fifth condition included high levels of perceptual load without degradation. The last two conditions were also included in Lavie and de Fockert's study. The load manipulation was based on that of Lavie and de Fockert. The target letter (N or X) was presented on an imaginary circle in one of eight possible locations. In the low load conditions there were no other non-target letters in the circle while in the high load condition the target was presented together with seven non-target letters (G, H, J, P, S, U, Y). All these different conditions are presented in Figure 1. If Lavie and de Fockert's findings were not merely due to the fact that only the target was degraded, the following results are expected: All low load conditions should result in considerable distractor interference with a larger interference in the degraded than non-degraded conditions, at least when only the target is degraded. There should be no distractor interference in the high load condition or at least the interference in this condition should be considerably smaller.

### Materials and methods

#### Participants

Eighteen naive observers, from the University of Haifa, with normal or corrected to normal vision participated in Experiment 1.

#### Stimuli

The target letter was an N or an X, presented in one of eight evenly spaced locations on an imaginary circle of letters (1.3° radius; 0.95° center to center distance of neighboring locations). The target was either presented together with other seven letters (G, H, J, P, S, U, Y) in the high-load-no-degradation (HLND) condition, or presented alone in all the other experimental conditions (Figure 1). The height and width of the letters on the circle were 0.6° × 0.4° of visual angle. The distractor letter was presented to the right or left of the imaginary circle, and its height and width were increased (1.0° × 0.55°) to control for the effect of eccentricity (Maylor and Lavie, 1998). The distractor was placed at an eccentricity of 3.2°. On one third of the trials the distractor was incompatible with the target (e.g., the target was the letter N and the distractor was the letter X); on another third of the trials the distractor was compatible with the target (e.g., both were the letter N); and on the rest of the trials the distractor was neutral (the letter P). The target and the distractor appeared equally often at each of their possible locations. In all conditions the letters were gray and the background was black (background luminance: 0.4 cd/m^2^). In the HLND and low-load-no-degradation (LLND) conditions the luminance of all the stimuli was 18.3 cd/m^2^. In the low-load-target-degraded (LLTD) condition the luminance of the target was 2 cd/m^2^ while the luminance of the distractor was 18.3 cd/m^2^. In the low-load-distractor-degraded (LLDD) condition the luminance of the distractor was 2 cd/m^2^ while the luminance of the target was 18.3 cd/m^2^. In the low-load-both-degraded (LLBD) condition the luminance of both target and distractor was 2 cd/m^2^.

#### Procedure

**Figure 2:**
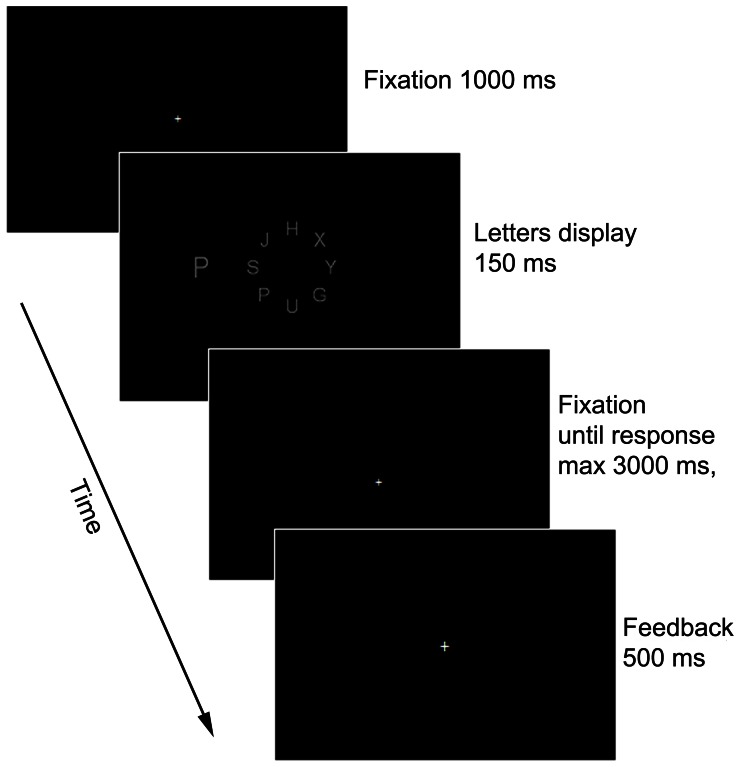
**A schematic illustration of a single trial in Experiment 1**.

The experiment took place in a dark room. Viewing distance was held fixed at 57 cm with a chin-rest. The task was to indicate as quickly and accurately as possible whether the target letter in the circle of letters was an X or an N (by clicking the N or the X keys), while ignoring the distractor. Each trial started with a 1000 ms fixation mark presented in the middle of the screen. To prevent eye movements, the letters display followed for a short duration of 150 ms (e.g., Mayfrank et al., 1987), and was replaced by a blank screen until the participant responded but no longer than 3000 ms (Figure 2). After responding, a 500 ms feedback was given: a “+” sign for a correct response, and a “−” sign for an incorrect response.

Each of the five different conditions (HLND, LLND, LLTD, LLDD, and LLBD) was presented in three different successive blocks of 96 trials each (total of 288 trials for each load-degradation condition, 96 for each compatibility condition). The presentation order of the five conditions was randomly chosen for each participant. Each participant viewed a total of 1440 experimental trials that were preceded by 30 practice trials.

### Results and discussion

**Figure 3:**
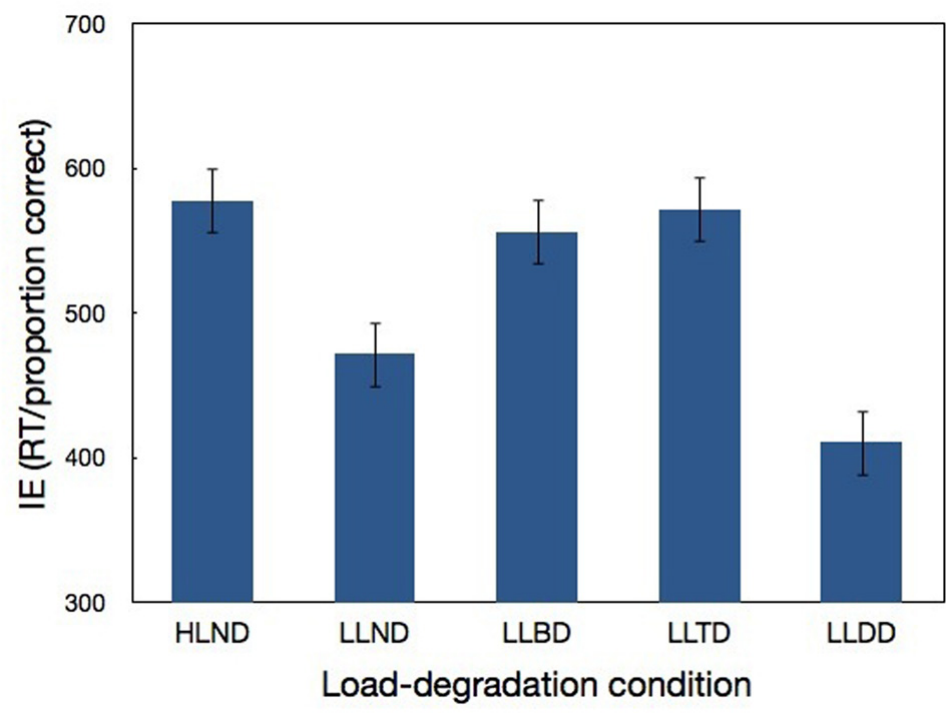
**Participants' IE scores (inverse efficiency: RT/proportion correct) in Experiment 1 as a function of load-degradation condition.** Error bars correspond to 1 SE.

The first block of each load-degradation condition served as practice and was excluded from the analysis. To take into consideration both accuracy and RT we calculated inverse efficiency (IE) scores by dividing the mean correct RT, for each condition of each participant, by the corresponding proportion correct (e.g., Townsend and Ashby, 1983). RTs shorter than 100 ms or longer than 2000 ms—0.18% from the total number of correct trials—were excluded from the calculation of mean RT. Like RT, higher IE scores indicate worse performance. In fact, IE scores are often referred to as “corrected RT” because they are considered as a measure of performance that circumvents possible criterion shifts or speed-accuracy tradeoffs (e.g., Townsend and Ashby, 1983; Murphy and Klein, 1998; Spence et al., 2001; Roder et al., 2007; Collignon et al., 2008). Table 1 presents the mean correct RT, accuracy (% correct) and IE scores for the various load-degradation and compatibility conditions. A two-way repeated measures ANOVA, load-degradation condition (HLND, LLND, LLBD, LLTD, or LLDD) × compatibility (neutral, incompatible, or compatible) was conducted on the IE scores^1^. The main effect of load-degradation condition was significant [*F*_(4, 68)_ = 20.03, *p* < 0.0001]. As can be seen in Figure 3 and Table 1, and confirmed by least significant differences (LSD) *post-hoc* analysis, performance in the non-degraded low-load condition (LLND) was significantly better (smaller IE scores; *p* < 0.0001) than in the high-load condition (HLND). This confirms that the manipulation of load was successful. The degradation manipulation was also successful, as decreasing the contrast of the target, either by itself (LLTD) or with the distractor (LLBD) resulted in a significantly worse performance (*p* < 0.0006) than when the target was not degraded (LLND, LLDD). Interestingly, a significant difference was also found between LLND and LLDD (i.e., worse performance when there was no degradation than when only the distractor was degraded; *p* < 0.02). This finding suggests that the conspicuity of the target relative to the distractor is indeed an important factor because when the relative conspicuity of the target was increased by decreasing only the contrast of the distractor, performance improved. The main effect of compatibility was also significant [*F*_(2, 34)_ = 63.68, *p* < 0.0001]. LSD *post-hoc* analysis indicated that performance in the incompatible condition was significantly worse than in either the neutral condition (*p* < 0.0001) or the compatible condition (*p* < 0.0001).

**Table 1:** **Mean correct RT, accuracy and IE scores (inverse efficiency = RT/accuracy) as a function of load-degradation and compatibility conditions in Experiment 1**.

**Load-degradation condition**	***RT* (ms) Distractor compatibility**				**Accuracy (%)** Distractor compatibility				***IE* scores Distractor compatibility**			
	***IC***	***C***	***N***	**Total**	***IC***	***C***	***N***	**Total**	***IC***	***C***	***N***	**Total**
HLND	542	538	537	539	92.4	93.6	95.1	93.7	588	577	567	577
LLND	466	424	427	439	90.3	94.9	95.5	93.6	518	447	449	471
LLBD	549	492	492	511	90.2	95.5	92.7	92.8	615	518	534	556
LLTD	552	500	514	522	87.6	94.5	93.3	91.8	634	529	552	572
LLDD	400	381	382	388	93.7	94.7	95.4	94.6	428	403	401	411
Total	502	467	471		90.8	94.6	94.4		557	495	501	

IC, incompatible condition; C, compatible condition; N, neutral condition.

**Figure 4:**
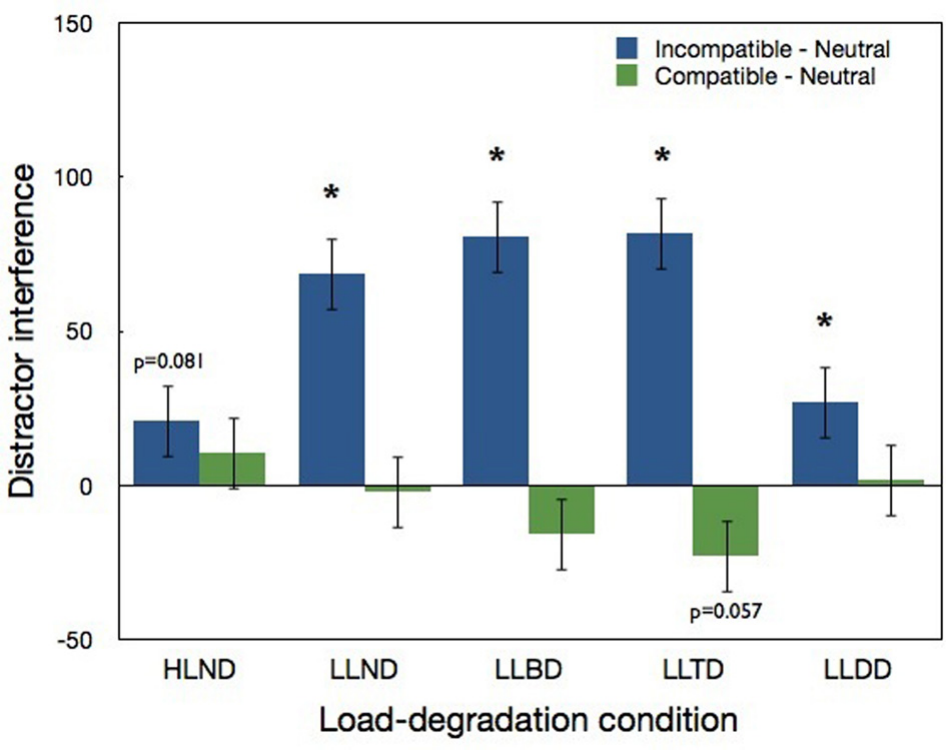
**Distractor interference (incompatible minus neutral) and distractor facilitation (compatible minus neutral) in Experiment 1 as a function of load-degradation condition.** “^*^” indicates a significant effect of the simple pairwise comparisons with neutral conditions. Error bars correspond to 1 SE.

Importantly, the two-way interaction between load-degradation condition and compatibility was significant [*F*_(8, 136)_ = 6.55, *p* < 0.0001]. Figure 4 shows that a significant distractor interference (incompatible—neutral) was found in all low load conditions (LLND, LLBD, LLTD: *p* < 0.0001; LLDD: *p* < 0.03). The fact that significant distractor interference was found in all the low load conditions regardless of degradation is in agreement with the perceptual load theory and with Lavie and de Fockert's (2003) claim that the degree of distractor interference does not depend on task difficulty. However, unlike Lavie and de Fockert's study, distractor interference in the target-degraded condition (LLTD) was not significantly higher than distractor interference in the non-degraded condition (LLND). Additionally, the interference effect in the LLBD and LLDD conditions indicates that the distractor was perceivable even when it was degraded, and the significantly larger interference in the LLTD than LLDD (*p* < 0.006) suggests that the conspicuity of the target relative to the distractor is important, because the interference was larger when the distractor was more conspicuous (LLTD) than when the target was more conspicuous (LLDD).

A marginally significant distractor interference (*p* = 0.081) was also found in the high-load condition (HLND). This interference is not consistent with the predictions of the perceptual load theory under the assumption that with high levels of load all the attentional resources were consumed by the central task. However, if the processing of central information with high levels of load did not consume all available resources, then the theory predicts some interference in the high load condition, though smaller than in the low load conditions. Indeed, the interference in the HLND condition was significantly smaller than in the LLND condition (*p* < 0.02) and LLTD and LLBD conditions (*p* < 0.003) but not LLDD condition. As for distractor facilitation (compatible—neutral), only a marginally significant effect (*p* = 0.057) was found in one condition—LLTD. This is consistent with Lavie's (1995) claim that the compatible condition is not optimally suited to explore the issue of distractor processing.

To sum, like the study of Lavie and de Fockert (2003) distractor interference was found even when the quality of the visual input was degraded. However, unlike their study degrading the target did not increase distractor interference. Interestingly, although distractor interference was found with degraded distractors, suggesting that the distractor was processed to a sufficient level, the interference was smallest when only the distractor was degraded (i.e., in comparison to the other low load conditions). Moreover, distractor interference was significantly larger when the distractor was more conspicuous in comparison to the target (LLTD) than when the target was more conspicuous in comparison to the distractor (LLDD). This finding suggests that the relative conspicuity of the target does play a role in determining the efficiency of the attentional selectivity. Finally, a marginally significant distractor interference was also found in the high load condition. This interference was smaller than the low load condition.

## Experiment 2

**Figure 5:**
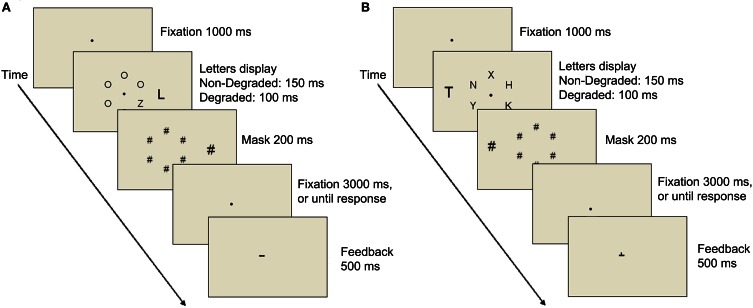
**A schematic illustration of a single trial in Experiment 2 with (A) low load display (B) high load display**.

This experiment tested whether similar results will emerge with a different manipulation of stimulus degradation and perceptual load. The degradation manipulation employed here involved exposure duration. Thus, the contrast of all the various stimuli was the same but in the degraded conditions the exposure duration of the stimuli was considerably shortened. Specifically, this experiment included two exposure duration conditions: a non-degraded condition in which exposure duration was 150 ms, and a degraded condition in which exposure duration was shortened to 100 ms. Each of these conditions included further manipulation of load and compatibility. The manipulation of compatibility was identical to that of Experiment 1, but the manipulation of load was different. In Experiment 1 perceptual load was manipulated by varying the set-size. Here, perceptual load was manipulated by changing the similarity between a target and non-target letters and the heterogeneity of the non-target letters (e.g., Lavie and Cox, 1997; Marciano and Yeshurun, 2011). In the low load condition, the imaginary circle included, in addition to the target, five homogeneous O's (Figure 5) while in the high load condition the other non-target letters were heterogeneous and shared features with the target (X, K, H, Y, V). If, as Lavie and de Fockert (2003) claim, perceptual load decreases distractor interference while sensory degradation increases it, then distractor interference should only be found with the low load conditions (or at least be larger than in the high load conditions), and a larger distractor interference should be found with the shorter exposure duration condition.

### Materials and methods

#### Participants

Twenty-four students from the University of Haifa took part in this experiment for monetary reward or course credit. All had normal or corrected to normal vision and all were naive to the purpose of the study. None of them participated in Experiment 1.

#### Stimuli

The stimuli of this experiment were similar to Experiment 1 except for the following: The target letter was either an N or a Z. Accordingly, in the compatible and incompatible conditions the distractor letter was also either an N or a Z. In the neutral condition the distractor was either an L or a T. There were only six possible locations on the imaginary circle of letters (2° radius; 1.7° center to center distance of neighboring locations). The target appeared equally often at each of the six possible locations. The other five letters were either all O's in the low load conditions or X, K, H, Y, and V in the high load conditions (Figure 5). The distractor was placed at an eccentricity of 4°. All the letters were black presented on a light gray background. The mask was composed of seven black # symbols located at each of the seven letters positions (six positions in the circle of letters and one position of the distractor letter). The size of the # symbol was identical to the size of the letter it masked (i.e., the # symbol that masked the distractor was larger).

#### Procedure

The procedure of this experiment was similar to Experiment 1 except for the following: The letters display was presented for a duration of 150 ms in the non-degraded condition and 100 ms in the degraded condition. The mask followed the letters display, and it was presented for 200 ms. Each block included 144 trials divided equally between the two exposure duration conditions (100 and 150 ms), and between the three compatibility conditions (compatible, incompatible, and neutral). The different exposure duration trials and the different compatibility trials were presented in random order within each block. The load conditions (low load and high load) were blocked. Similar to Forster and Lavie (2007), the order of the load blocks was fixed for all participants: low, high, low, high, high, low, low, high, high, low. Each participant performed 1440 trials, 720 of each exposure duration condition and 480 of each condition of load.

### Results and discussion

The first two blocks served as practice and were excluded from the analysis. As in Experiment 1, we calculated IE scores for each condition of each participant, with the same RT exclusion criterion (excluding 0.29% from the total number of correct trials). These IE scores were submitted to a three-way repeated measures ANOVA, load (low vs. high) x exposure duration (150 vs. 100 ms) × compatibility (neutral, incompatible, or compatible). The means of RT, accuracy and IE scores for all the conditions are presented in Table 2. All three main effects were significant: performance was better (smaller IE scores) with low than high load levels [*F*_(1, 23)_ = 108.93, *p* < 0.0001], longer than shorter exposure durations [*F*_(1, 23)_ = 29.09, *p* < 0.0001], and was best in the compatible condition and worst in the incompatible condition [*F*_(2, 46)_ = 39.47, *p* < 0.0001]. Thus, the manipulations of load and degradation employed in this experiment were successful. The two-way interaction between compatibility and exposure duration was also significant [*F*_(2, 46)_ = 4.66, *p* < 0.02]. LSD *post-hoc* analysis indicated that distractor interference (incompatible—neutral) was significant in both exposure durations (*p* < 0.0001), yet it was larger with the 100 ms than 150 ms duration.

**Table 2:** **Mean correct RT, accuracy and IE scores (inverse efficiency = RT/accuracy) as a function of load-degradation and compatibility conditions in Experiment 2**.

**Load-degradation condition**	***RT* (ms) Distractor compatibility**				**Accuracy (%)** Distractor compatibility				***IE* scores Distractor compatibility**			
	***IC***	***C***	***N***	**Total**	***IC***	***C***	***N***	**Total**	***IC***	***C***	***N***	**Total**
Low load 100	624	600	608	611	83.4	91.9	89.1	88.1	762	659	688	703
High load 100	665	674	676	672	65.1	75.6	74.8	71.8	1033	904	917	951
Low load 150	611	578	600	596	87.4	92.7	91.6	90.6	704	629	658	664
High load 150	701	694	699	698	72.3	79.1	78	76.5	982	889	913	928
Total	650	637	646		77.1	84.8	83.3		870	770	794	

IC, incompatible condition; C, compatible condition; N, neutral condition.

**Figure 6:**
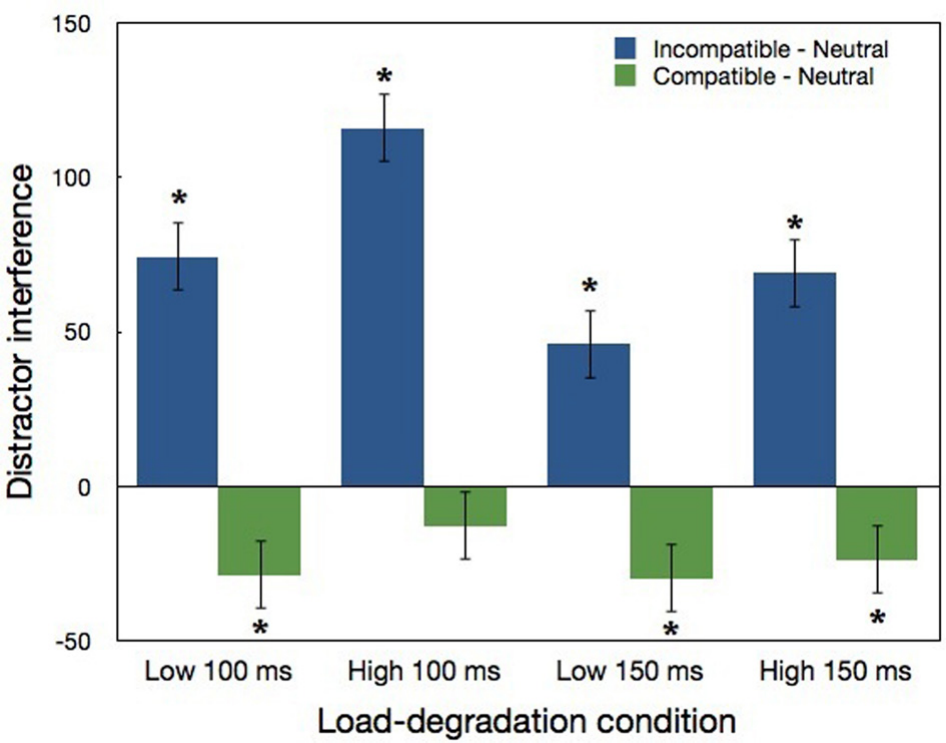
**Distractor interference (incompatible minus neutral) and distractor facilitation (compatible minus neutral) in Experiment 2 as a function load-degradation condition.** “^*^” indicates a significant effect of the simple pairwise comparisons with neutral conditions. Error bars correspond to 1 SE.

The three-way interaction between load, exposure duration, and compatibility was not significant. Nevertheless, LSD *post-hoc* analysis was performed because of its theoretical importance. As can be seen in Figure 6 and Table 2, in the low load conditions significant distractor interference was found in both 150 and 100 ms duration (*p* < 0.0002). Thus, as predicted based on Lavie and de Fockert (2003), increasing task difficulty by reducing the exposure duration did not eliminate the interference induced by the incompatible distractor. Also in accordance with Lavie and de Fockert, the magnitude of the interference was larger with marginal significance (*p* = 0.095) in the degraded 100 ms condition than in the non-degraded 150 ms condition. Notwithstanding the confirmation of Lavie and de Fockert's assertion regarding distractor interference and task difficulty, the basic prediction of the perceptual load theory was not confirmed because with both exposure durations significant distractor interference was found in the high load condition (*p* < 0.0001). Moreover, this high load interference was not smaller in magnitude, as may be expected according to the perceptual load theory. Indeed, the magnitude of the distractor interference in the high load condition of the 150 ms duration did not differ significantly from the interference of the corresponding low load condition, and was in fact larger (*p* < 0.02) in the high than low load conditions of the 100 ms duration. Regarding distractor facilitation (compatible—neutral), a significant difference (*p* < 0.04) was found for all the load-degradation conditions apart from the high load 100 ms condition.

To sum, like the study of Lavie and de Fockert (2003) and Experiment 1 of this study, distractor interference was found even when the quality of the visual input was degraded. Additionally, degrading the display resulted in larger distractor interference that was marginally significant. A significant distractor interference was found in the high load conditions. This is similar to Experiment 1 in which a marginally significant high load interference emerged, however in the current experiment this interference was not smaller than the interference of the low load condition with 150 ms duration and even larger with the 100 ms duration. Thus, these results are not in line even with the weaker version of the perceptual load theory that allows for a smaller interference with high levels of load. This unexpected distractor interference is further explored in Experiments 3, 4.

## Experiment 3

The stimuli and procedure of Experiment 2 (particularly the 150 ms condition) were very similar to Experiment 4 in a previous study of ours (Marciano and Yeshurun, 2011). Still, in that experiment no interference was found in the high load condition while in Experiment 2 of this study we found such high-load distractor interference that was similar or even larger than the low-load interference. One methodological difference that might have led to this discrepancy is the presence of masks in Experiment 2. That is, in Experiment 2 masks followed the letter display with both exposure durations, but in Experiment 4 of Marciano and Yeshurun's study, which only included exposure duration of 150 ms, there was no such backward masking. The mask was added in Experiment 2 because the main degradation manipulation in that experiment was exposure duration shortening, and a mask is required to ensure brief presentation. Could the addition of a mask explain the emergence of distractor interference with high levels of load?

According to the perceptual load theory the addition of a mask should not have mattered—there should be no distractor interference (or a smaller interference) in the high load condition regardless of the presence or absence of a mask. This is because according to the theory the attentional selection is strictly passive, stemming from the exhaustion of the available processing capacity imposed by the higher perceptual load. The addition of a mask after the offset of the letter display should not affect the availability of resources, and therefore should not affect the magnitude of distractor interference. In contrast, with a more active view of the attentional selectivity, in which the lack of distractor interference reflects an active inhibition, the mask might matter if we assume that this inhibition requires time to exert its effect. When the stimuli are masked there might not be enough time to develop full inhibition and distractor interference emerges. This explanation gains some support from the fact that distractor interference in the shorter (100 ms) high load condition of Experiment 2 was significantly larger (*p* < 0.005) than that of the longer (150 ms) high load condition. Hence, when there was less time for inhibition to evolve a larger interference was observed. If indeed the emergence of distractor interference with high load levels is due to the presence of the mask, then once the mask is removed the interference should disappear or at least decrease considerably. To test this prediction, this experiment was identical to Experiment 2 apart from not including backward masking.

### Materials and methods

#### Participants

Eighteen students from the University of Haifa took part in this experiment for monetary reward or course credit. All had normal or corrected to normal vision and all were naive to the purpose of the study. None of them participated in the previous experiments.

#### Stimuli and procedure

The stimuli and procedure of this experiment were identical to Experiment 2 except for the fact that a mask did not follow the letter display.

### Results and discussion

As in previous experiments, we calculated IE scores for each condition of each participant, with the same RT exclusion criterion (excluding 0.21% from the total number of correct trials, after the exclusion of the first two blocks that served as practice). These IE scores were submitted to a three-way repeated measures ANOVA, load (low vs. high) × exposure duration (150 vs. 100 ms) × compatibility (neutral, incompatible, or compatible). The means of RT, accuracy and IE scores for all the conditions are presented in Table 3. The main effect of load was significant [*F*_(1, 17)_ = 81.16, *p* < 0.0001]; poorer performance (larger IE scores) were found with high than low load conditions. The main effect of compatibility was also significant [*F*_(2, 34)_ = 107.93, *p* < 0.0001]. Performance was best in the compatible condition and worst in the incompatible condition. The main effect of exposure duration was not significant (*p* = 0.58), suggesting that without a backward mask the manipulation of exposure duration is not effective.

**Table 3:** **Mean correct RT, accuracy and IE scores (inverse efficiency = RT/accuracy) as a function of load-degradation and compatibility conditions in Experiment 3**.

**Load-degradation condition**	***RT* (ms) Distractor compatibility**				**Accuracy (%)** Distractor compatibility				***IE* scores Distractor compatibility**			
	***IC***	***C***	***N***	**Total**	***IC***	***C***	***N***	**Total**	***IC***	***C***	***N***	**Total**
Low load 100	612	567	574	584	93.3	95.7	95.9	95	656	594	599	616
High load 100	726	710	713	716	86.9	91.2	89.9	89.4	841	782	797	807
Low load 150	607	569	581	586	94.4	96.8	96.1	95.8	642	589	605	612
High load 150	735	712	709	719	87.3	90.9	90.3	89.5	846	787	789	807
Total	670	640	644		90.5	93.7	93.1		746	688	698	

IC, incompatible condition; C, compatible condition; N, neutral condition.

**Figure 7:**
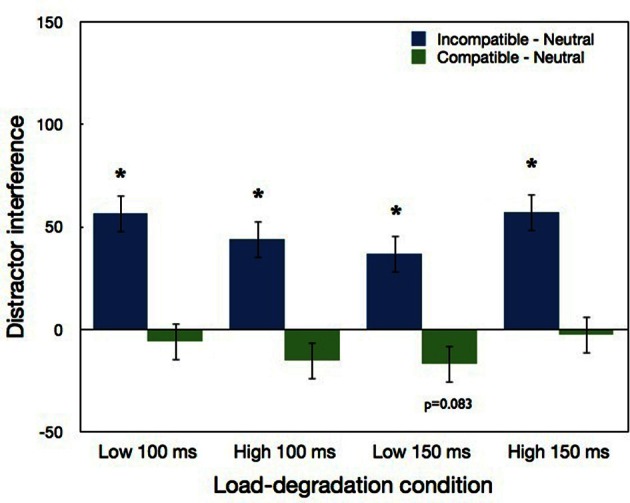
**Distractor interference (incompatible minus neutral) and distractor facilitation (compatible minus neutral) in Experiment 3 as a function load-degradation condition.** “^*^” indicates a significant effect of the simple pairwise comparisons with neutral conditions. Error bars correspond to 1 SE.

The three-way interaction between load, exposure duration and compatibility was not significant, still LSD *post-hoc* comparisons were analyzed due to their theoretical importance. As can be seen in Figure 7, in the low load conditions distractor interference was significant in both the 100 ms condition (*p* < 0.0001), and the 150 ms condition (*p* < 0.0004). As in our previous experiments, the significant distractor interference found in the harder low load condition (i.e., 100 ms exposure duration) is consistent with Lavie and de Fockert's (2003) claim that task difficulty *per se* is not the reason for the lack of interference with high levels of load. Also similar to Experiment 1, the interference in the 100 ms low load condition was not significantly larger than in the 150 ms low load condition. Finally, in contrast to the predictions of the perceptual load theory but similar to our previous experiments, significant distractor interference also emerged in the high load conditions (*p* < 0.0001), and with both duration conditions the magnitude of the interference did not differ significantly in the high vs. low load conditions. Thus, the observed high load interference was not smaller in magnitude, as may be expected according to the weaker version of the perceptual load theory. Regarding distractor facilitation, a marginally significant difference (*p* = 0.083) was found only for the low load 150 ms condition.

To sum, this experiment replicated the main findings of our two previous experiments: shortening the exposure duration did not decrease distractor interference but also did not increase it. However, unlike Experiment 2, the two exposure duration conditions did not differ significantly, suggesting that without a backward mask the manipulation of degradation via shortening of exposure duration is not effective. Importantly, the goal of this experiment was not to test whether the manipulation of exposure duration without a backward mask is an effective way to degrade the visual input, but rather to test whether the presence of a mask is a critical factor for the emergence of distractor interference under high levels of load. Thus, the critical outcome of this experiment is the fact that a significant distractor interference was found in both high load conditions, and this interference was statistically similar to the interference of the low load conditions. Thus, the exclusion of the mask did not eliminate or even reduce distractor interference with high levels of load. This issue is further explored in Experiment 4.

## Experiment 4

The results of Experiment 3 suggest that the discrepancy between our current Experiment 2 and Experiment 4 of our previous study (Marciano and Yeshurun, 2011) is not due to the presence of a backward mask because Experiment 3 did not include a mask, yet an interference was found in its high load condition. The only other methodological difference between the experiments is the fact that in Experiment 2 the variable of exposure duration was mixed. Hence, in Experiment 4 of Marciano and Yeshurun (2011) all the trials within a single block were similar apart from the compatibility of the distractor, while in Experiment 2 they also differed considerably in terms of the duration of the main letters display. The perceptual load theory, due to its passive view of attention, holds no place for effects that are related to such within-block variance. But a more active view of the attentional selectivity can accommodate effects that are due to within-block variability. This is because larger within-block variance creates more uncertainty regarding the demands of the upcoming trial, and this uncertainty may encourage the participants to adopt a different selection strategy than when there is less within-block variability. In this experiment we tested whether the emergence of distractor interference with high load levels was indeed an outcome of large within-block variability. To that end the variable of duration in this experiment was blocked. If the distractor interference in the high load condition found in Experiments 2, 3 is due to the fact that the variable of duration was mixed within a block, no such interference should be found here or at least its magnitude should decrease considerably.

### Materials and methods

#### Participants

Eighteen students from the University of Haifa took part in this experiment for monetary reward or course credit. All had normal or corrected to normal vision and all were naive to the purpose of the study. None of them participated in the previous experiments.

#### Stimuli and procedure

The stimuli and procedure of this experiment were identical to Experiment 2 except for the fact that the variable of exposure duration was blocked. The experimental meeting included two consecutive sessions, each session included six blocks of trials, all with the same exposure duration—either 100 or 150 ms. The order of the duration sessions was counterbalanced across participants. Each block included 144 trials divided equally between the three compatibility conditions (compatible, incompatible, and neutral). Overall, each participant performed 1728 trials, 864 of each exposure duration condition and 576 of each load condition.

### Results and discussion

As in previous experiments, we calculated IE scores for each condition of each participant, with the same RT exclusion criterion (excluding 0.36% from the total number of correct trials, after the exclusion of the first two blocks in each duration session that served as practice). These IE scores were submitted to a three-way repeated measures ANOVA, load (low vs. high) x exposure duration (150 vs. 100 ms) × compatibility (neutral, incompatible, or compatible). The means of RT, accuracy and IE scores for all the conditions are presented in Table 4. The main effect of load was significant [*F*_(1, 17)_ = 100.95, *p* < 0.0001]; performance was poorer (larger IE scores) with high than low load conditions. The main effect of compatibility was also significant [*F*_(2, 34)_ = 59.87, *p* < 0.0001]. Performance was best in the compatible condition and worst in the incompatible condition. The main effect of exposure duration was practically significant [*F*_(1, 17)_ = 4.38, *p* = 0.0516]; performance was better with the longer than shorter exposure duration. The two-way interaction between compatibility and exposure duration was also significant [*F*_(2, 34)_ = 6.25, *p* < 0.005]: distractor interference was significant in both exposure durations (*p* < 0.0007), yet it was larger with the 100 ms than 150 ms duration. Distractor facilitation was significant in 100 ms condition (*p* < 0.02) but not in the 150 ms condition.

**Table 4:** **Mean correct RT, accuracy and IE scores (inverse efficiency = RT/accuracy) as a function of load-degradation and compatibility conditions in Experiment 4**.

**Load-degradation condition**	***RT* (ms) Distractor compatibility**				**Accuracy (%)** Distractor compatibility				***IE* scores** Distractor compatibility			
	***IC***	***C***	***N***	**Total**	***IC***	***C***	***N***	**Total**	***IC***	***C***	***N***	**Total**
Low load 100	657	629	643	643	80.3	92.7	88.7	87.2	845	682	733	753
High load 100	679	675	690	681	64.4	76.5	75.1	72	1085	891	934	970
Low load 150	665	633	642	647	88.7	94.9	92.5	92	752	668	695	705
High load 150	693	697	689	693	73.1	80.9	79.6	77.9	962	872	880	905
Total	674	659	666		76.6	86.3	84		911	778	811	

IC, incompatible condition; C, compatible condition; N, neutral condition.

**Figure 8:**
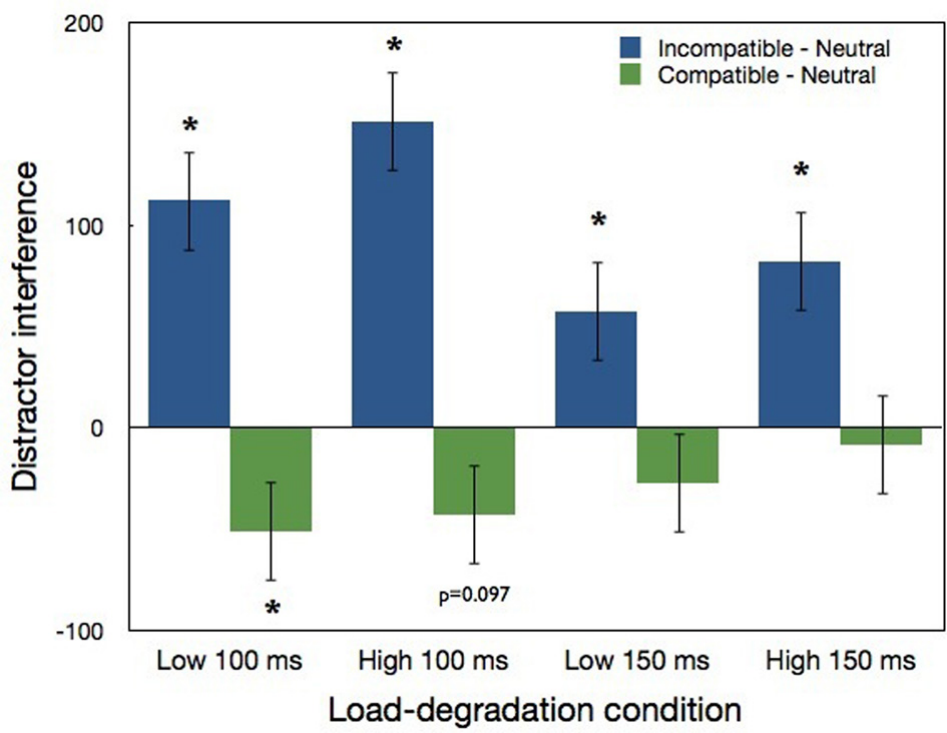
**Distractor interference (incompatible minus neutral) and distractor facilitation (compatible minus neutral) in Experiment 4 as a function load-degradation condition.** “^*^” indicates a significant effect of the simple pairwise comparisons with neutral conditions. Error bars correspond to 1 SE.

The three-way interaction between load, exposure duration and compatibility was not significant. Nevertheless we performed LSD *post-hoc* analysis because of its theoretical importance. As can be seen in Figure 8 and in Table 4, in the low load condition significant distractor interference was found in both 150 ms (*p* < 0.03) and 100 ms durations (*p* < 0.0001). However, similar to our previous experiments, but unlike Lavie and de Fockert (2003), the magnitude of this interference did not differ significantly between the two duration conditions. Also similar to our previous experiments, significant distractor interference in the high load condition emerged with both exposure durations (*p* < 0.003). Moreover, the magnitude of the distractor interference in the high load conditions of both durations did not differ significantly from the interference of the low load conditions. Regarding distractor facilitation, a significant difference (*p* < 0.05) was found for the low load 100 ms condition, and marginally significant difference (*p* = 0.097) was found for the high load 100 ms condition.

To sum, this experiment replicated the main findings of our previous experiments: degrading the quality of the visual input did not decrease the low load distractor interference, but also did not increase it. In addition, a significant distractor interference was found in the high load conditions, and this interference was statistically similar to the interference of the low load condition. Thus, blocking the duration variable did not eliminate or even reduce distractor interference with high levels of load.

## General discussion

This study examined the effects of degrading the quality of the visual stimulus on distractor interference. To that end we degraded the stimuli in two different manners. In Experiment 1 we reduced the stimuli contrast and in Experiments 2–4 we shortened exposure duration. The outcome was similar in all four experiments: degrading the quality of the visual input did not eliminate distractor interference. This finding is similar to Lavie and de Fockert (2003), however in that study only the target was degraded. This latter fact raises the possibility that distractor interference in Lavie and de Fockert's study “survived” degradation because in addition to mere degrading the quality of the target the degradation changed the conspicuity relationships between the target and distractor—making the distractor considerably more conspicuous than the target. Here, we degraded both relevant and non-relevant stimuli, and still found that distractor interference persists. However, Lavie and de Fockert also found that degrading the target under low levels of load increased the magnitude of distractor interference. They, therefore, concluded that while increasing perceptual load decreases distractor interference, increasing sensory load (i.e., degrading the sensory input) increases distractor interference. We found very little evidence in support of the conclusion that increasing sensory load increases interference. In almost all of the cases explored here (apart from a marginally significant effect in Experiment 2) there was no significant difference between the degraded and non-degraded conditions in terms of distractor interference. This discrepancy between our data and that of Lavie and de Fockert may be due to the conspicuity issue mentioned above. That is, in their study the degradation also made the target less conspicuous in comparison to the distractor and this resulted in increased interference from the more salient distractor. The issue of conspicuity will be further discussed below. Given the data accumulated thus far, it seems that the only solid conclusion one can make regarding stimulus degradation (or sensory load), is that it plays only a minor role, if at all, in determining the efficiency of the attentional selectivity.

Benoni and Tsal (2012) have recently reached a similar conclusion. They did not attempt to control for target distractor conspicuity relationships, as we did here. Hence, like Lavie and de Fockert (2003), their displays included only degradation of the target. Instead, they controlled for the effect of dilution. As these authors demonstrated in several studies (e.g., Benoni and Tsal, 2010; Tsal and Benoni, 2010), adding neutral letters to a low load display (i.e., diluting the distractor) eliminates distractor interference. This finding suggests that the typical lack of distractor interference in the high load condition may not be due to increase in demand for resources due to high levels of load, but to the fact that the display also includes neutral letters that dilute the distractor. In their recent study, Benoni and Tsal (2012) examined the effect of degrading the quality of the target when the effects of dilution are controlled for. Across their two experiments they compared the magnitude of distractor interference between displays of low-load-low-dilution (i.e., no neutral letters), low-load-high-dilution (i.e., with neutral letters and a cue marking target position), and high-load-high-dilution (i.e., with neutral letters but without the cue), each with and without target degradation (contrast and size reduction). They found that the main factor that determined the magnitude of distractor interference was not sensory load (or perceptual load), but rather the presence of neutral letters. Thus, Benoni and Tsal's findings are consistent with the assertion that sensory degradation has only a minor effect on the attentional selectivity.

**Figure 9:**
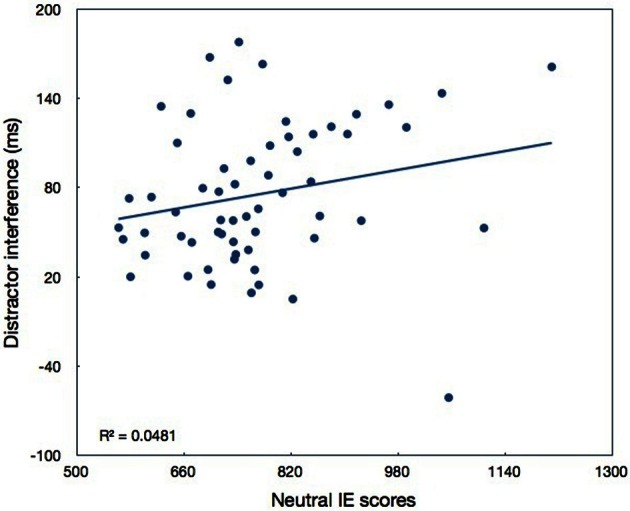
**Distractor interference as a function of performance in the neutral condition of Experiments 2–4**.

The motivation stated in Lavie and de Fockert (2003) to examine sensory degradation was to rule out an alternative explanation claiming that the lack of distractor interference in the high load condition is due to the fact that the task in this condition was harder. That is, that the factor determining the attentional selectivity is not perceptual load but task difficulty. By showing that task difficulty in the degraded condition was high, but distractor interference was not reduced, Lavie and de Fockert (2003) could conclude that this alternative explanation is not valid. Our data also suggest that task difficulty *per se* is not a critical factor, because we often found large interference in the hard high load conditions. To examine more carefully the effect of task difficulty by itself, we plot in Figure 9 distractor interference as a function of performance (IE scores) in the neutral condition for each of the participants in Experiments 2–4. We assume here that performance in the neutral condition is the most uncontaminated measure of task difficulty we have in this study. Also, we combined the last three experiments because of their high methodological similarity. As can be seen in Figure 9, there is only a weak relationship between the magnitude of distractor interference and performance in the neutral condition. Specifically, there is a marginally significant positive correlation (*r* = 0.22, *n* = 60, *p* = 0.0923): distractor interference is larger with worse performance^2^. Thus, this correlation is in the opposite direction to that suggested as an alternative explanation. In any case, this correlation can only account for 4.8% of the variance in distractor interference. When one outlier is taken out of the analysis this correlation reaches statistical significance (*r* = 0.364, *n* = 59, *p* < 0.005). Still, even without the outlier, performance in the neutral condition (task difficulty) can only account for 13.3% of the variance in distractor interference. This leads to the conclusion that task difficulty *per se* does not play an important role in our ability to select relevant information, and the minor role it may have is in fact in the opposite direction to that assumed before, as larger interference was found with harder tasks.

Interestingly, perceptual load also did not emerge as a critical factor. In all of our experiments a reliable distractor interference was found in the high load condition. This interference was marginally significant in Experiment 1 and significant in Experiments 2–4. This high load interference was often of the same magnitude as the low load interference. Similar high load interference also emerged in Experiments 1–3 of our previous study (Marciano and Yeshurun, 2011). In that study we attributed the high load interference to uncertainty regarding the spatial location of the distractor, because when this uncertainty was reduced from 10 possible locations to two possible locations (Experiments 1–3 vs. Experiment 4 of that study), the high load interference was eliminated. However, in all of our current experiments there were only two possible distractor locations. Hence, the high load interference found here could not be attributed to high spatial uncertainty. These findings suggest, therefore, that although low level of uncertainty regarding the location of the distractor may be a necessary condition to allow efficient distractor exclusion, it is not a sufficient condition. Regardless of spatial uncertainty, the findings of the current study as well as our previous study suggest that the efficiency of the attentional selectivity does not depend on perceptual load, at least not in the way described by the perceptual load theory, because inefficient selectivity (i.e., distractor interference) is sometimes found only with low levels of load, but sometimes it is also found with high levels of load. That is, perceptual load is not a strong predictor of selection efficiency.

Indeed, the core tenets of the perceptual load theory were already challenged in the past [see Khetrapal (2010) for a review]. Several studies have demonstrated that efficient selection (i.e., the lack of distractor interference) is possible even under conditions of low perceptual load (e.g., Paquet and Craig, 1997; Johnson et al., 2002; Eltiti et al., 2005; Tsal and Benoni, 2010). More relevant to our current study are prior demonstrations of distractor interference under high load conditions (e.g., Chen, 2003; Theeuwes et al., 2004; Eltiti et al., 2005; Tsal and Benoni, 2010; Benoni and Tsal, 2012). Theeuwes et al. (2004), for instance, found that when high and low load conditions were intermixed within the same block, distractor interference was found in both conditions. They suggested that low perceptual load can bring about broad attentional processing that carries over to subsequent high load trials. This inter-trial influences account, however, cannot explain the high load interference found here because in all of the current experiments the load manipulation was blocked. Chen (2003) found that when the non-relevant and relevant information were part of the same object the levels of perceptual load did not modulate the degree of interference. This finding is also not applicable to the current study, because the relevant and non-relevant information in the current study always belonged to different objects. Eltiti et al. (2005) claim that the efficiency of selective attention depends not only on perceptual load but also on the saliency of the target and distractor in comparison to the neutral items. They found that increasing the target and distractor saliency by using a target that is slightly larger than the neutral letters and employing onset distractors, results in an interference effect even under high perceptual load. They suggested that because the target and the distractor were the most salient items both captured attention and this resulted in interference. However, because the target in our Experiments 2–4 was not more salient than the other letters, this saliency interpretation of the high load interference cannot account for our entire data set. Still, some of our findings in Experiment 1 may be due to saliency differences between target and distractor (see more below). Finally, as described above, diluting the effect of the distractor by adding to the display neutral letters that share features with the target and distractor, eliminates distractor interference (e.g., Tsal and Benoni, 2010; Benoni and Tsal, 2012). Most relevant to the current findings, these authors also found that when the low-load diluted condition is compared to the high-load condition larger distractor interference is observed in the high load than dilution condition. This is consistent with our findings of reliable high load interference, and further suggests that when the high load interference is compared to the interference in a diluted low load condition, rather than the typical not-diluted low load condition, the high load interference may be even larger than the low load interference.

Unlike the factors discussed above, the conspicuity of the target relative to the distractor does seem to play a role in determining the efficiency of the attentional selectivity. This is because distractor interference in Experiment 1 was significantly larger when the distractor was more conspicuous in comparison to the target (i.e., when only the target was degraded—LLTD) than when the target was more conspicuous in comparison to the distractor (i.e., when only the distractor was degraded—LLDD). This finding is consistent with Eltiti et al. (2005) claim that the saliency of the target is an important factor. They made the target more salient than the distractor by presenting the target as onset and the distractor as offset, and demonstrated that this eliminated distractor interference. In our Experiment 1 target relative saliency was manipulated via contrast reduction and the outcome was similar—considerably smaller interference with the less salient distractor. In the same line, reducing the saliency of the distractor by adding neutral items that share features with the distractor considerably reduces distractor interference (e.g., Benoni and Tsal, 2010, 2012; Tsal and Benoni, 2010). Nonetheless, the relative conspicuity of the target cannot be the only factor mediating attentional selectivity because the relative conspicuity of the target was identical in our current Experiments 2–4 and Experiment 4 of Marciano and Yeshurun (2011) as well as Experiment 1 of Lavie and Cox (1997), yet distractor interference was absent in the previous experiments (the latter two experiments) but present in the current experiments.

Given these different patterns of results obtained in highly similar experiments (in terms of their methodological details) there seems to be a need for reconsideration of theories of attentional selectivity suggested thus far. At this point we can only speculate that the diverse outcomes are due to complex interactions between multitude of factors that encourage the participants to adopt different strategies regarding distractor exclusion. For instance, maybe if the task is too easy the participants do not bother investing resources in inhibiting the distractor because a reasonable level of performance can be attained without such inhibition. But when the task is moderately hard, distractor inhibition is “worth” the investment because perceiving the distractor might have a more detrimental effect on performance. Still, if the task is particularly hard the participants may not have spare resources to invest in inhibition. Such non-monotonic effects of task difficulty could obscure the true function of this factor. In addition, if the target is more salient than the distractor, distractor inhibition may not be needed or fewer resources may be required to prevent interference. This may also alter the selection strategy adopted by the participants, independently from task difficulty. Above all, there might be factors that are related to individual differences (e.g., working memory capacity) that play a major role in determining the selection strategy adopted by the participants. Such factors may be the ones responsible for the different patterns of results obtained for similar experiments. Considerable additional research is required to shed light on this issue. Nevertheless, it is hard to see how the current and previous outcomes can fit into the passive view of the attentional selectivity offered by the perceptual load theory. It seems much more consistent with an active view of selectivity, in which distractor interference is prevented via an active inhibition of non-relevant stimuli (Marciano and Yeshurun, 2011). Such an active view of attentional selectivity is consistent with Torralbo and Beck's (2008) study, in which distractor interference was found only when the target and other non-relevant items were presented to different hemifields. That is, evidence of selectivity was found only when there were nearby non-relevant items that compete over neuronal representation. Such competitive interactions may encourage the participants to adopt a more strict selectivity strategy. Torralbo and Beck suggested that these active biasing processes operate to improve the representation of the target, but it is quite likely that both enhancement of the relevant information and inhibition of non-relevant information may take place simultaneously when a need to exclude the distractor arises.

To conclude, neither stimulus degradation, established via contrast reduction (Experiment 1) or brief exposure duration (Experiments 2–4), nor perceptual load affected distractor interference in a consistent way. Distractor interference could be found with or without stimulus degradation and under low or high levels of load. These findings suggest that both factors do not have a critical role in determining our ability to ignore non-relevant items. A more complex model of attentional selectivity is required to account for the diversity of results reported thus far.

### Conflict of interest statement

The authors declare that the research was conducted in the absence of any commercial or financial relationships that could be construed as a potential conflict of interest.

## Footnotes

^1^The same analysis was performed separately on mean correct RT and accuracy. In all the experiments reported here, these separate analyses and the analysis of IE scores lead to similar conclusions. The RT and accuracy analyses are provided as supplementary material.

^2^Similar analyses were performed on the RT and accuracy measures. With RTs there was no significant correlation (*r* = −0.15, *n* = 60, *p* = 0.26), even when one outlier participant was taken out of the analysis (*r* = −0.13, *n* = 59, *p* = 0.34). With accuracy there was also no significant correlation when all participants were included (*r* = −0.2, *n* = 60, *p* = 0.12), but when one outlier was taken out of the analysis this correlation reached statistical significance (*r* = −0.33, *n* = 59, *p* < 0.02). Note that the negative correlation in the case of accuracy is in the same direction as that found with IE scores—interference is larger with worse performance.
