# Supplementary material for: Degraded stimulus visibility and the effects of perceptual load on distractor interference
Source: Front Psychol. 2013 May 29;4:289. doi: 10.3389/fpsyg.2013.00289 (PMC3665929; doi:10.3389/fpsyg.2013.00289)
Supplement: Supplementary file 1 [file Presentation1.PDF]

Supplementary material

Experiment 1

The first block of each load-degradation condition served as practice and was excluded from the analysis. RTs shorter than 100 ms or longer than 2000 ms were also excluded from the analysis (0.18% from the total number of correct trials). A two-way repeated measures ANOVA, load-degradation condition (HLND, LLND, LLBD, LLTD, or LLDD) x compatibility (neutral, incompatible, or compatible) and the corresponding least significant differences (LSD) post-hoc analysis were conducted on mean correct RT and accuracy data. The means of all the conditions are presented in Table 1.

***RT analysis*:** The main effect of load-degradation condition was significant [$F(4, 68)=22.86$, $p<0.0001$]; all pairwise comparisons were significant ($p<0.03$). RTs were longer in the high load condition (HLND) than in any of the low load conditions. Decreasing the contrast of the target, either by itself (LLTD) or with the distractor (LLBD), prolonged RTs compared to low load conditions in which the target was not degraded (LLND, LLDD). Longer RTs were found when only the target was degraded (LLTD) than when both target and distractor were degraded (LLBD) and when there was no degradation (LLND) than when only the distractor was degraded (LLDD). The main effect of compatibility was also significant [$F(2, 34)=30.52$, $p<0.0001$]. RTs in the incompatible condition were significantly longer than RTs in either the neutral condition ($p<0.0001$) or the compatible condition ($p<0.0001$). The two-way interaction was significant [$F(8, 136)=4.91$, $p<0.0001$]. In all load-degradation conditions, excluding HLND, significant distractor interference (incompatible vs. neutral) was found (LLND, LLBD, LLTD: $p<0.0001$; LLDD: $p<0.04$). Distractor interference in the target degraded condition (LLTD) was not higher than distractor interference in the non degraded condition (LLND). The interference in the LLTD was significantly larger than LLDD ($t(17)=2.22$, $p<0.05$). Distractor facilitation (compatible vs. neutral) did not reach statistical significance in any of the conditions.

***Accuracy analysis*:** The main effect of load-degradation condition was significant [$F(4, 68)=2.59$, $p<0.05$]; accuracy in the LLTD condition was significantly lower ($p<0.05$) than either HLND, LLND, or LLDD conditions. In addition accuracy in the LLBD condition was significantly lower than in the LLDD condition ($p<0.05$). The main effect of compatibility was also significant [$F(2, 34)=26.70$, $p<0.0001$]. Accuracy in the incompatible condition was significantly lower than in the other compatibility conditions ($p<0.0001$). As with RT, the two-way interaction was significant [$F(8, 136)=3.75$, $p<0.0006$]. In all load-degradation conditions, excluding LLDD, significant distractor interference was found (HLND: $p<0.02$; LLND: $p<0.0001$; LLBD: $p<0.03$; LLTD: $p<0.0001$). The interference in the HLND condition was smaller with marginal significance than in the LLTD condition ($t(17)=1.76$, $p=0.096$), but did not differ from the LLND condition. Similar to the RT data, distractor interference in the target degraded condition (LLTD) was not significantly higher than distractor interference in the non degraded condition (LLND). Also similar to the RT data is the finding of a significantly larger distractor interference in the LLTD than LLDD condition ($t(17)=2.46$, $p<0.03$). A significant distractor facilitation was only found in the LLBD condition.

Experiment 2

The first two blocks served as practice and were excluded from the analysis. RTs shorter than 100 ms or longer than 2000 ms were also excluded from the analysis (0.29% from the total number of correct trials). A three-way repeated measures ANOVA, load (low vs. high) x exposure duration (150 ms vs. 100 ms) x compatibility (neutral, incompatible, or compatible) and the corresponding LSD post-hoc analysis were conducted on mean correct RT and accuracy data. The means of all the conditions are presented in Table 2.

***RT analysis*:** The main effect of load was significant [$F(1, 23)=35.43$, $p<0.0001$]; RTs were longer with high than low load conditions. The main effect of compatibility was also significant [$F(2, 46)=5.68$, $p<0.007$]. RTs in the incompatible condition were longer than RTs in the neutral condition, which were longer than RTs in the compatible condition. The two-way interaction between load and compatibility was significant [$F(2, 46)= 7.58$, $p<0.002$]. A significant distractor interference (incompatible vs. neutral) was found in the low load condition ($p<0.002$), but not in the high load condition. The two-way interaction between load and exposure duration was also significant [$F(1,23)=30.74$, $p<0.001$]. This interaction is due to the fact that in the low load condition RTs were significantly longer with the 100 ms than 150 ms condition ($p<0.0001$). In contrast, in the high load condition, RTs were significantly faster with the 100 ms than 150 ms condition ($p<0.0001$). Although the three-way interaction was not significant, it was further analyzed due to its theoretical importance. In the low load condition distractor interference was significant in the 100 ms condition ($p<0.02$), and close to significant in the150 ms condition ($p=0.055$). In the high load condition with 100 ms exposure duration a marginally significant facilitation from an incompatible distractor was found ($p=0.079$), but this specific condition was 'contaminated' by speed-accuracy tradeoff and its outcomes are therefore questionable. Regarding distractor facilitation (compatible vs. neutral), a significant difference ($p<0.001$) was found only for the low load 150 ms condition.

***Accuracy analysis*:** All three main effects were significant: Accuracy was higher with low than high load levels [$F(1, 23)=194.69$, $p<0.0001$], longer than shorter exposure durations [$F(1, 23)=72.85$, $p<0.0001$]; and was highest in the compatible condition and lowest in the incompatible condition [$F(2, 46)=43.14$, $p<0.0001$]. The two-way interaction between compatibility and exposure duration was also significant [$F(2, 46)=8.84$, $p<0.001$]. Distractor interference was significant in both exposure durations ($p<0.0001$), yet it was larger with the 100 ms than 150 ms duration. Similarly, distractor facilitation was significant in the 100 ms condition ($p<0.005$) but only marginally significant in the 150 ms condition ($p=0.07$). The two-way interaction between load and exposure duration was marginally significant [$F(1, 23)=3.7$, $p=0.07$]. In agreement with the RT data, accuracy in the low load conditions was significantly higher with the 150 ms than 100 ms duration ($p<0.0001$). In contrast to the RT data, accuracy in the high load conditions was also significantly higher with the 150 ms than 100 ms duration ($p<0.0001$). Thus, the relatively fast RTs in the high-load 100 ms condition is probably due to speed-accuracy-tradeoffs. The three-way interaction was not significant. Still, it was further analyzed because of its theoretical importance. In the low load conditions significant distractor interference was found in both 150 ms and 100 ms duration ($p<0.0001$). The magnitude of the interference in these two conditions did not differ significantly. With both exposure durations significant distractor interference was also found in the high load condition ($p<0.0001$). The interference did not differ significantly between the high and low load conditions of the 150 ms duration, but it was larger with marginal significance ($t(23)=-1.93$, $p=0.066$) in the high than low load conditions of the 100 ms duration. Regarding distractor facilitation, a significant difference ($p<0.005$) was found only for the low load 100 ms condition.

Experiment 3

The first two blocks served as practice and were excluded from the analysis. RTs shorter than 100 ms or longer than 2000 ms were also excluded from the analysis (0.21% from the total number of correct trials). A three-way repeated measures ANOVA, load (low vs. high) x exposure duration (150 ms vs. 100 ms) x compatibility (neutral, incompatible, or compatible) and the corresponding LSD post-hoc analysis were conducted on mean correct RT and accuracy data. The means of all the conditions are presented in Table 3.

***RT analysis*:** The main effect of load was significant [$F(1, 17)=92.83$, $p<0.0001$]; RTs were longer with high than low load conditions. The main effect of compatibility was also significant [$F(2, 34)=59.57$, $p<0.0001$]. RTs in the incompatible condition were longer than RTs in the neutral condition, which were longer than RTs in the compatible condition. The two-way interaction between load and compatibility was significant [$F(2, 34)=4.35$, $p<0.03$]. A significant distractor interference (incompatible vs. neutral) was found in both load conditions (low: $p<0.0001$; high: $p<0.0005$), but it was smaller in the high load condition. Although the three-way interaction was not significant, it was further analyzed due to its theoretical importance. In the low load conditions distractor interference was significant in both the 100 ms condition ($p<0.0001$), and the 150 ms condition ($p<0.0008$). Distractor interference also emerged in the high load conditions. The interference was significant with the 150 ms exposure duration ($p<0.0007$), and marginally significant with the 100 ms exposure duration ($p=0.086$). With the 100 ms duration, the interference in the low load condition was larger than that of the high load condition with close to significance difference ($t(17)= 2.10$, $p=0.051$). However, with the 150 ms duration, there was no significant difference between the interference in the high and low load conditions. Regarding distractor facilitation (compatible vs. neutral), a marginally significant difference ($p<0.088$) was found only for the low load 150 ms condition.

***Accuracy analysis*:** The main effect of load was significant: Accuracy was higher with low than high load levels [$F(1, 17)=44.46$, $p<0.0001$]. The main effect of compatibility was also significant: Accuracy was highest in the compatible condition and lowest in the incompatible condition [$F(2, 34)=24.24$, $p<0.0001$]. The three-way interaction was not significant. Nevertheless it was further analyzed because of its theoretical importance. In the low load condition significant distractor interference was found in both 150 ms ($p<0.04$) and 100 ms duration ($p<0.002$). The magnitude of the interference in these two conditions did not differ significantly. With both exposure durations significant distractor interference was also found in the high load condition ($p<0.0004$). The magnitude of the distractor interference in the high load conditions of both durations did not differ significantly from the interference of the low load conditions. Regarding distractor facilitation, a marginally significant difference ($p=0.091$) was found only for the high load 100 ms condition.

Experiment 4

The first two blocks in each duration session served as practice and were excluded from the analysis. RTs shorter than 100 ms or longer than 2000 ms were also excluded from the analysis (0.36% from the total number of correct trials). A three-way repeated measures ANOVA, load (low vs. high) x exposure duration (150 ms vs. 100 ms) x compatibility (neutral, incompatible, or compatible) and the corresponding LSD post-hoc analysis were conducted on mean correct RT and accuracy data. The means of all the conditions are presented in Table 4.

***RT analysis*:** The main effect of load was significant [$F(1, 17)=12.81$, $p<0.003$]; RTs were longer with high than low load conditions. The main effect of compatibility was also

significant [$F(2, 34)=14.27$, $p<0.0001$]. RTs in the incompatible condition were significantly longer than RTs in the neutral condition ($p<0.05$), which were significantly longer than RTs in the compatible condition ($p<0.04$). The two-way interaction between load and compatibility was significant [$F(2, 34)= 11.43$, $p<0.0002$]. A significant distractor interference (incompatible vs. neutral) was found only in the low load condition ($p<0.001$). The three-way interaction was not significant but it was nevertheless analyzed due to its theoretical importance. In the low load conditions distractor interference was significant in the 150 ms condition ($p<0.003$), and marginally significant in the100 ms condition ($p=0.0634$). The interference of these two conditions did not differ significantly. There was no significant interference in any of the high load conditions. Regarding distractor facilitation (compatible vs. neutral), a significant facilitation was found for the high load 100 ms condition ($p<0.04$) and marginally significant for the low-load 100 ms condition ($p=0.051$).

***Accuracy analysis*:** All three main effects were significant: Accuracy was higher with low than high load levels [$F(1, 17)=82.90$, $p<0.0001$], longer than shorter exposure durations $F(1, 17)=27.34$, $p<0.0001$]; and was highest in the compatible condition and lowest in the incompatible condition [$F(2, 34)=75.72$, $p<0.0001$]. All the relevant pairwise comparisons were significant ($p<0.02$). The two-way interaction between compatibility and exposure duration was significant [$F(2, 34)=9.07$, $p<0.0008$]: distractor interference was significant in both exposure durations ($p<0.0003$), yet it was larger with the 100 ms than 150 ms duration. Distractor facilitation was significant in 100 ms condition ($p<0.04$) but not in the 150 ms condition. The two-way interaction between load and compatibility was also significant [$F(1, 17)=3.45$, $p<0.05$]: distractor interference was significant in both load conditions ($p<0.0001$), but it was larger with the high than low load conditions. Although the three-way interaction was not significant it was further analyzed because of its theoretical importance. In the low load condition significant distractor interference was found in both 150 ms ($p<0.04$) and 100 ms durations ($p<0.0001$). This interference was significantly larger in the degraded low load condition ($t(17)=2.35$, $p<0.04$). With both exposure durations, significant distractor interference in the high load condition ($p<0.0007$) emerged. Moreover, the magnitude of the high load interference of both durations did not differ significantly from the interference of the low load conditions. Regarding distractor facilitation, a significant difference ($p<0.03$) was found only for the low load 100 ms condition.
